# Physical properties and water absorption kinetics of three varieties of *Mucuna* beans

**DOI:** 10.1038/s41598-021-85087-8

**Published:** 2021-03-09

**Authors:** Ajibola B. Oyedeji, Olajide P. Sobukola, Ezekiel Green, Oluwafemi A. Adebo

**Affiliations:** 1 Department of Biotechnology and Food Technology, Faculty of Science, University of Johannesburg Doornfontein Campus, P. O. Box 17011, Gauteng, 2028 South Africa; 2 Department of Food Science and Technology, College of Food Science and Human Ecology, Federal University of Agriculture, Ogun State, P.M.B. 2240, Abeokuta, Nigeria

**Keywords:** Biophysics, Engineering

## Abstract

The physical properties and water absorption kinetics of three varieties of *Mucuna* beans (*Mucuna pruriens, Mucuna rajada and Mucuna veracruz*) were determined in this study. Physical properties including length, width, thickness, geometric mean diameter, sphericity, porosity, bulk density, area, volume and one thousand seed mass were calculated while hydration kinetics was studied by soaking *Mucuna* beans in water at 30 °C, 40 °C and 50 °C and measuring water uptake at 9 h interval. Peleg’s equation was used to model the hydration characteristics and Arrhenius equation was used to describe the effect of temperature on Peleg’s rate constant k_1_ and to obtain the activation energies for soaking. Significant variations were observed in almost all the physical properties of the different varieties, however, there were no significant differences (p < 0.05) in their thicknesses and bulk densities. The effectiveness of fit of Peleg’s model (R^2^) increased with increase in soaking temperature. Peleg’s rate constant k_1_ decreased with increase in soaking temperature while k_2_ increased with temperature increase. Activation energies of *Mucuna pruriens, Mucuna rajada and Mucuna veracruz* were 1613.24 kJ/mol, 747.95 kJ/mol and 2743.64 kJ/mol, respectively. This study provides useful information about the properties of three varieties of *Mucuna* beans that could be of importance to processors and engineers for process design and optimization.

## Introduction

*Mucuna* belongs to the leguminous plant genera consisting of about 100 species of climbing vines and shrubs of the family *Fabaceae*^1^. It is classified as an underutilized legume because to its limited use due to some of its properties, such as hardness and prolonged cooking time. Other limiting attributes of *Mucuna* include methionine deficiency, presence of anti-nutritional factors such as trypsin inhibitors, thioglucosides and haemagluttinins. It also contains 3,4 dihydroxy-L-phenylalanine (L-DOPA), an anti-nutritional and toxic chemical that must first be removed before the utilization of the seeds^2^. Some species of *Mucuna* beans have their pod surfaces covered in coarse hairs which contains ‘mucunanin’, a proteolytic enzyme which when in contact with the skin causes severe irritation and itchy blisters^3^.

Adebowale et al.^4^ reported a range of 2.74–5.88% moisture content for six varieties of *Mucuna* beans; *Mucuna pruriens, M. cochinchinensis M. rajada, M. veracruz white, M. veracruz mottle* and *M. deeringeana* while carbohydrate contents were fairly high (43.7–49.7%). Although starch was the dominant carbohydrate, some reducing and non-reducing sugars were also found in the seeds. Crude proteins content ranged between 33.2 and 38.4% while potassium (356–433 mg/100 g) was the most abundant of the valuable minerals and trace elements in the seeds. Also, the seeds have high calcium and phosphorus contents. Although, methionine and cysteine are limiting amino acids, protein digestibility of the seeds is high (81.3–85.5%) and this confers potential usefulness on *Mucuna* beans, like other popularly consumed or utilized legumes.

The knowledge of physical properties of *Mucuna* beans, like those of other seeds and grains, is important in designing equipment for handling and processing operations such as harvesting, separation, aeration, drying and storage. Hence, cleaning, grading and separation equipment are designed specifically on the basis of the physical properties of seeds to be handled. Amin et al.^5^ noted that the practical utility of the structural design of bio-processing machines depends on the basic physical properties of seeds to be processed. There are documented reports on the handling and processing of agricultural materials through mechanical, optical and other techniques^6–8^, but little is known about the basic physical properties of *Mucuna* species. These information are important not only to engineers, but also to breeders, processors and food scientists and other professionals^9^.

Water absorption characteristics of *Mucuna* seeds, as a unit operation preceding dehulling and milling, is considered crucial in its food processing considerations^10^. Therefore, understanding the water uptake kinetics of *Mucuna* is important since it has direct effects on the subsequent processing operations and final product quality^11^. Since soaking temperature and time are key factors determining the amount of water absorbed by food materials^12^, it is necessary to characterize and optimize the water absorption phenomenon as a function of time and temperature^11^. Rehydration kinetics of different beans and peas had already been studied by Turhan et al.^11^, Solomon^13^, and Kaptso et al.^14^, however, information about the water absorption kinetics of *Mucuna* beans is yet to be properly documented.

Considering the high quality nutritional composition of *Mucuna* and its high potential for use in food processing, there is a need to study its physical properties and the water absorption kinetics to make information about its suitability for processing available to food scientists and processors and further popularize its utilization. Hence, this study sought to determine some physical properties and time–temperature water uptake relationships in three varieties of *Mucuna* beans.

## Materials

Three varieties of healthy *Mucuna* seeds were obtained from the International Institute of Tropical Agriculture, (IITA), Ibadan, Nigeria. These varieties are *M. pruriens IRZ, M. veracruz* black and *M. rajada*. Samples were handpicked to remove broken seeds and ensure homogeneity of seeds in terms of size, shape and dimensions.

## Methodology

### Determination of the physical properties

Physical properties of *Mucuna* seeds such as linear dimensions, sphericity, geometric diameter, surface area, volume, seed densities (true and bulk), porosity and one-thousand seeds mass were determined using the methods described by Baryeh^15^, with slight modifications for different properties. The seeds were at the same condition of temperature and moisture before these physical properties were determined. Each of the physical properties were determined in triplicates.

### Linear dimensions

For each variety, 20 seeds were selected at random and their individual lengths (L) which is the distance between the seed eye and the opposite, width (W) and thickness (T) which are seed major and minor diameters respectively were determined using the micrometer screw gauge (436-25 M Starret, Brazil), with ± 0.01 mm accuracy allowance. From the information of their length, width and thickness, the following parameters were calculated according to the relations given by Mohsenin (2020);

### Sphericity (Φ)

Sphericity of the seeds (mm^−1^) was determined using the relation:

$$ \Phi = \frac{{\left( {\text{L WT}} \right)^{1/3} }}{{\text{L}}} $$

where L—length (mm), W—width (mm), T—thickness (mm).

### Geometric mean diameter (D_g_)

Geometric mean diameter was determined using the relation:

$$ {\text{D}}_{{\text{g}}} = ({\text{LWT}})^{1/3} $$

### Surface area (S)

The surface area S (mm^2^) was calculated using the relation:

$$ {\text{S }} = \pi {\text{D}}_{{\text{g}}}^{2} $$

where $\pi$ = 3.142 and D_g_ is the geometric diameter.

### Volume (V_g_)

The volume V_g_ (mm^3^) was calculated using the relation:

$$ {\text{V}}_{{\text{g}}} = \frac{{{\uppi } \cdot {\text{WTL}}^{2} }}{{6\left[ {2{\text{L}} - \left( {{\text{WT}}} \right)^{1/2} } \right]}} $$

### Seed true density (ρ_g_)

True density ρ_g_ (g/ml) was determined by water displacement method. 300 ml of water was placed in a 1000 ml graduated measuring cylinder and then, 30 g of each variety was poured into the water in the measuring cylinder. The amount of water displaced was then recorded. Short duration of measurement was ensured to prevent water absorption by kernel as stipulated by Aviara et al*.*^16^.

$$ \rho_{{\text{g}}} = \frac{{{\text{weight of seeds }}\left( {\text{g}} \right)}}{{{\text{volume of water displaced }}\left( {{\text{ml}}} \right)}} $$

### Seed bulk density (ρ_b_)

Bulk density ρ_b_ (g/ml) was determined with modifications from the previous studies of Okezie and Bello^17^.

$$ \rho_{{\text{b}}} = \frac{{{\text{mass of seeds in the measuring cylinder }}\left( {\text{g}} \right)}}{{{\text{volume of the measuring cylinder }}\left( {{\text{ml}}} \right)}} $$

### Porosity (ε)

The porosity ε of the seed is the fraction of the space in the bulk seed which is not occupied by the grain. It was determined using the relationship stated by Baryeh^15^;

$$ \varepsilon = \frac{{{\uprho }_{g} - {\uprho }_{b} }}{{{\uprho }_{g} }} \times 100 $$

### One thousand seed mass

Five hundred seeds of each variety were counted and weighed. The weights obtained were then multiplied by two (2) to obtain the one thousand seed mass.

### Rehydration of *Mucuna* seeds for water absorption studies

The rehydration method described by Sobukola and Abayomi^18^ was adopted in the treatment of *Mucuna* seeds in this study. The initial moisture contents of seeds of each variety were first determined by weighing 5 g of dry grounded kernels in electronic weighing balance (Metler Toledo 1227190560, China, with ± 0.0001 mm accuracy allowance) and transferring into a hot air oven at 105 °C for 3 h. Thereafter, water uptake of the seeds was determined by soaking 10 g of samples of seeds of each variety in 250 ml beakers containing 100 ml distilled water. Soaking temperatures were set to 30 °C, 40 °C and 50 °C in water bath (Labcon 23782, Maraisburg, South Africa). These temperatures were chosen to simulate the soaking conditions used in households. The beakers, water and seeds were first equilibrated to the required soaking temperature before rehydration experiments in each case. At one-hour interval, the immersed seeds were removed from water, dried of excess surface water using paper towel and weighed in electronic weighing balance (Metler Toledo 1227190560, China). At each interval, moisture uptake of the seeds was determined as the difference between the weight of the dry seeds and the soaked seeds. Three experimental replicates were performed for each variety and the samples were drawn every hour from 1 to 9 h^11,14^.

### Modelling of water uptake

Peleg’s equation^19^, which is an empirical model, was used in modeling the water uptake pattern of the *Mucuna* seeds. The model states:

$$ U_{t} = U_{o} + { }\frac{{\text{t}}}{{{\text{k}}_{{1{ }}} + {\text{k}}_{2} {\text{t}}}} $$

where $U_{o}$ is the initial moisture content (%), $U_{t}$ is the mean moisture content (%) at time t, $k_{1 }$ is the Peleg’s rate constant (h%^−1^) and $k_{2}$ is the Peleg’s capacity constant (%^−1^). In line with this model, the equilibrium moisture content $U_{e}$ at prolonged soaking time is given by the following equation which indicates the association between k_2_ and water absorption capacity of the seeds^19^.

$$ U_{e} = U_{o} + { }\frac{1}{{{\text{k}}_{2} }} $$

The linearized form of Peleg equation was fitted into the water absorption data to describe hydration characteristics at each temperature^13^. The same approach had been used in earlier studies by Sopade et al.^20^ and Abu-Ghannam and McKenna^21^:

$$ \frac{{\text{t}}}{{U_{t} - U_{o} }} = {\text{k}}_{1} + {\text{k}}_{2} {\text{t}} $$

### Determination of activation energy

The temperature dependence of the reciprocal of $k_{1}$ is expressed by the Arrhenius relationship:

$$ \ln \frac{1}{{{\text{k}}_{1} }} = { }\ln {\text{k}}_{{\text{o}}} + \frac{{{\text{E}}_{{\text{a}}} }}{{{\text{RT}}}} $$

where $E_{a}$ is the activation energy (kJ/(kg mol)), R is gas constant (8.318 kJ/mol K) and T is the absolute soaking temperature (K). $E_{a}$ was calculated from the slope of ln $\frac{1}{{k_{1} }}$ versus $\frac{1}{t}$, while $k_{o}$ (%min^−1^) is the intercept^22^. Data fit 8.2 (Oakdale engineering 1995–2006) package was used for the modeling of the water absorption pattern of the seeds.

### Statistical analysis

Experimental data were obtained in triplicates. Data were statistically analysed using one-way analysis of variance (ANOVA) and means were separated using Duncan Multiple Range test using the Statistical Package for Social Science (SPSS) version 16 (Chicago IL, USA).

## Results and discussions

### Physical properties of *Mucuna* beans

Some physical properties of the three varieties of *Mucuna* beans such as linear dimensions, sphericity, porosity, area, volume, true and bulk densities, and the one thousand seed mass were determined (Table 1). These properties were determined without varying the initial moisture content of the beans. The initial moisture content of *M. pruriens* was 11.62 ± 0.14%, *M. rajada* was 11.26 ± 0.89% and *M. veracruz* was 11.05 ± 0.66% and this may be due to physiological differences in varieties. These values are slightly higher than what was reported by Ezeagu et al.^23^ for these varieties obtained from the same source, but at different times. The results of linear dimensions of *Mucuna* beans were different from those of lupin as reported by Solomon^13^ who found the length, width, thickness and geometric mean diameter of lupin to be 9.68 mm, 8.70 mm, 4.83 mm and 7.41 mm, respectively. There were significant variations (p < 0.05) between the length, width and geometric mean diameter of the varieties of *Mucuna* beans but there was no significant variation in the thickness of these varieties. These linear dimensions will help in designing the aperture size of bean handling machinery^24^. Sphericity of *Mucuna* beans in this study ranged from 0.92 to 0.97 mm^−1^. This result shows that *Mucuna* bean is almost perfectly spherical and so could be treated as spheres for the prediction of drying behaviour. Sphericity values were similar to what was obtained for *Bambara* nuts^15^ and green wheat^24^. No significant difference was observed in the true densities of *Mucuna* beans (1.32–1.33 g/ml), while bulk densities were significantly different, ranging between 0.81 and 0.87 g/ml.

**Table 1:** Physical properties of different varieties of *Mucuna* beans.

Physical properties	*M. pruriens*	*M. rajada*	*M. veracruz*
Length (mm)	12.24 ± 0.90^a^	9.75 ± 0.57^a^	10.79 ± 1.29^a^
Width (mm)	17.14 ± 1.13^c^	10.47 ± 0.66^a^	13.80 ± 1.32^b^
Thickness (mm)	7.47 ± 0.80^ab^	8.29 ± 0.52^b^	6.90 ± 0.49^a^
Geometric mean diameter (mm)	11.35 ± 0.12^c^	8.56 ± 0.40^a^	9.92 ± 0.09^b^
Sphericity (mm^−1^)	0.97 ± 0.02^b^	0.95 ± 0.01^b^	0.92 ± 0.01^a^
Porosity (%)	37.33 ± 2.08^b^	39.33 ± 1.50^b^	34.00 ± 1.00^a^
True density (g/ml)	1.33 ± 0.005^a^	1.33 ± 0.00^a^	1.32 ± 0.02^a^
Bulk density (g/ml)	0.81 ± 0.02^a^	0.87 ± 0.02^b^	0.83 ± 0.02^a^
Surface area (mm^2^)	408.53 ± 0.40^c^	281.10 ± 0.08^a^	310.22 ± 0.11^b^
Volume (mm^3^)	742.53 ± 0.34^c^	424.38 ± 0.11^a^	458.39 ± 0.02^b^
One thousand seed mass (g)	960.00 ± 28.84^c^	672.67 ± 11.54^b^	575.33 ± 8.08^a^

Means with the different alphabets are significantly different at P < 0.05 along the same row.

Although, there were significant differences between the sphericity, porosity and bulk densities of these varieties, true densities were not significantly different (Table 1). Adequate knowledge of these properties will help in the separation and transportation of *Mucuna* beans by hydrodynamics^25^. Porosity of *Mucuna* varieties in this study ranged between 34.00 and 39.33%. The result of the surface area and volume were found to be 281.10–408.53 mm^3^ and 424.38–742.53 mm^3^, respectively. These high values have the potential to provide good aeration and water vapour diffusion properties during deep bed drying. One thousand seed mass of the three varieties was found to range between 575 and 960 g, with *M. pruriens* having the highest weight. Significant variations were also observed for one thousand seed masses among individual varieties. It follows that the individual mass of every seed of each variety could be calculated or predicted for further engineering considerations.

### Water absorption kinetics

Using a linearized Peleg’s regression model, the values obtained from the water uptake studies of the three varieties of *Mucuna* were used to derive the values of Peleg’s constants k_1_ (rate constant) and k_2_ (capacity constant). Peleg’s model has been widely adopted and used for predicting water sorption kinetics in many seeds, especially for short-time rehydration procedures (usually below 24 h of soaking)^11^. Similar methods have been used to obtain the values of k_1_ and k_2_ by other authors^13,22,26^. The results obtained are as shown in Table 2.

**Table 2:** Water absorption characteristics of *Mucuna* beans using Peleg’s model.

Variety	Temperature (°C)	k_1_ (h%^−1^)	k_2_ (%^−1^)	R^2^
*M. pruriens*	30	4.0912	0.3524	0.18
	40	2.2039	0.6750	0.79
	50	0.5792	0.7814	0.91
*M. rajada*	30	3.3450	0.4191	0.25
	40	0.8390	0.8043	0.95
	50	0.6593	0.8571	0.96
*M. veracruz*	30	7.1619	-0.1171	0.01
	40	2.7733	0.5192	0.57
	50	0.9197	0.7038	0.88

### Water absorption phenomena

The rate of water absorption, often referred to as the driving force, depends on the difference between the moisture content at saturation and at a given time^13^. As hydration proceeded for all the varieties, the moisture content increased, leading to a decrease in the absorption rate and driving force. The process ceased when the seeds attained the equilibrium moisture content^27^. The shape of the absorption curves obtained reflects the degree of hardness of the shell of the beans. The three varieties of *Mucuna* beans studied presented features of hard seed coats and seed hardening which is characterized by insoluble carboxyl groups of pectins^14^ and lignifications through cross linking of phenols in the seed coat, preventing water penetration during cooking^28^. Of the three varieties, *M. rajada* had the fastest rate of water absorption while *M. veracruz* had the least. This could be attributed to the fact that the seed coat of *M. rajada* is the softest while that of *M. veracruz* is the hardest. Also, the activation energy (E_a_ in kJ/mol) of *M. rajada* is the least while that of *M. veracruz* is the highest (Table 3). This implies that higher soaking temperatures are required to achieve proper soaking. During the experiment, as soaking temperature increased, the rate of water absorption also increased, leading to the attainment of the equilibrium moisture content faster than when lower soaking temperature was used. This behaviour could be linked to the higher rate of water diffusion at a higher temperature. Similar trends have been reported for different peas and beans by Turhan et al*.*^11^ Abu-Ghannam and McKenna^21^, Hung et al*.*^29^, and Kader^30^.

**Table 3:** Activation energy of hydration of each variety of *Mucuna* beans.

Variety	Temperature (°C)	E_a_ (kJ/mol)
*M. pruriens*	30–50	1631.24
*M. rajada*	30–50	747.95
*M. Veracruz*	30–50	2743.64

### Modelling of the water uptake kinetics of *Mucuna* beans

**Figure 1:**
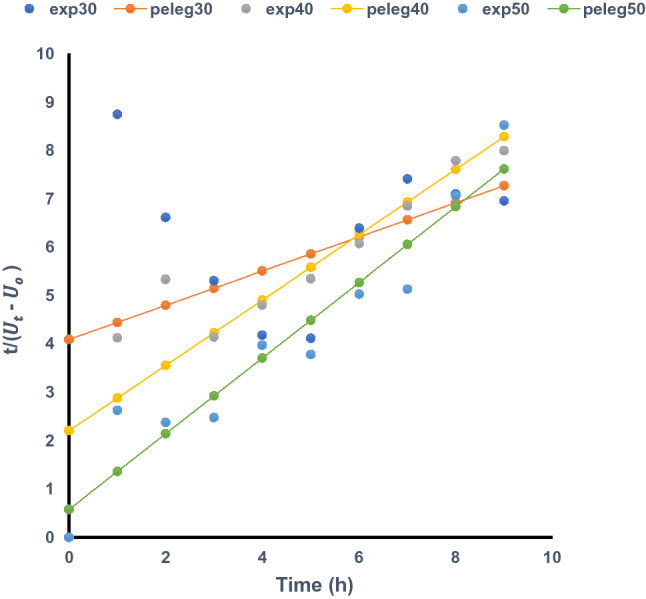
Experimental and Peleg (predicted) water uptake of *Mucuna pruriens* at different temperatures.

**Figure 2:**
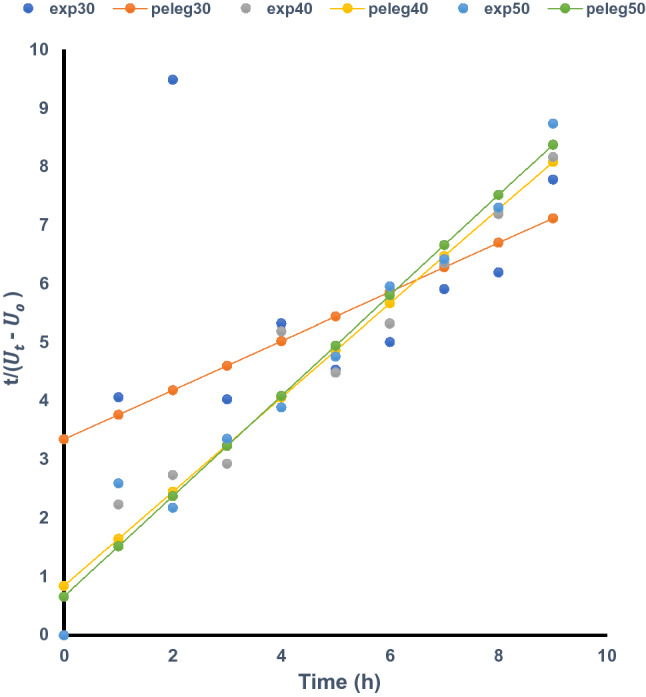
Experimental and Peleg (predicted) water uptake of *Mucuna rajada* at different temperatures.

**Figure 3:**
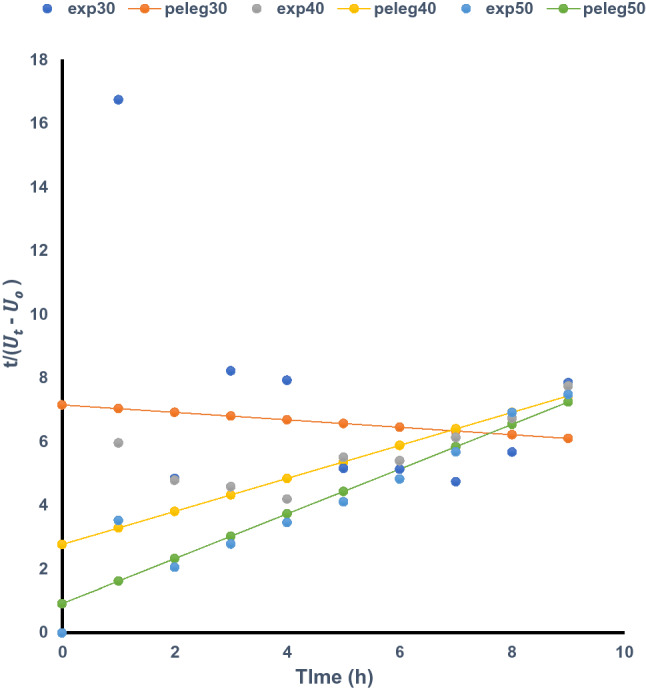
Experimental and Peleg (predicted) water uptake of *Mucuna veracruz* at different temperatures. SPSS software url: ibm.com/products/spss-statistics. Data fit software url: https://datafit.software.informer.com/8.2/.

The fit of Peleg’s equation to the experimental data during the soaking period of the *Mucuna* bean varieties at the stipulated soaking temperatures are presented in Figs. 1, 2 and 3. Rehydration is an important unit operation in food processing as it affects the effectiveness of other stages of processing and the overall quality of the final product^11,14^. The results of the regression models fitted to the experimental data at the hydration temperatures are shown in Table 2. The coefficient of determination (R^2^) values ranged between 0.01 and 0.96. Low R^2^ values were obtained in some instances for each variety and could be attributed to hardness of the seeds, characterized by slow water diffusion rate, especially at lower soaking temperatures, leading to a slow rate of water absorption by the seeds. This trend was observed for the three varieties when the soaking temperature was 30 °C. Higher R^2^ values were obtained at increased temperatures of 40 and 50 °C soaking temperatures. A similar trend was also noticed in the rehydration kinetics of *Bambara* in the study of Jideani and Mpotokwane^28^. Peleg’s model had a poor fit for the experimental data at 30 °C and better fits at 40 and 50 °C. The seed coat of *M. veracruz* was the hardest of the three *Mucuna* varieties studied. The low R^2^ value obtained at the lowest soaking temperature for *M. veracruz* could be an indication that water uptake at this temperature is extremely low, as a result of the hardness of seed coats of this variety. This is further justified by the considerable increase in R^2^ value upon the increase in soaking temperature. This is also depicted by the value of Peleg capacity constant (k_2_) which measures the capacity of the seeds to absorb water at the soaking temperature. It could thus be inferred that the fit of Peleg’s model to the water uptake kinetics of *Mucuna* beans increases with increase in soaking temperature.

### Peleg’s capacity constant k_2_ and the final moisture content

From the values of k_2_ obtained for each variety of *Mucuna* beans (Table 2), it was observed that k_2_ increased as the soaking temperature increased. The highest values of k_2_ was obtained for *M. rajada*. The increase in k_2_ as temperature increased, was previously reported by Solomon^13^ who found that k_2_ is related to water absorption capacity in that water absorption capacity decreased with increase in temperature. There are varying reports on the dependence of constant k_2_ on processing temperature by many authors. Lopez et al.^31^ reported a decrease in k_2_ in hazelnut and hazelnut-kernel as the soaking temperature increased. Also, decrease in k_2_ was reported in amaranth grain^27^ and wheat products^22^ with increase in soaking temperature. On the other hand, some studies have shown that k_2_ is unaffected by temperature as reported by Sopade and Obekpa^32^ for peanuts, cowpea and soybeans, Sopade et al.^20^ for maize and sorghum, Hung et al.^29^ for chicken and field peas and Abu-Ghannam and McKenna^21^ for blanched red kidney beans. Soaking at 50 °C brought about reduction of the time required to reach the maximum moisture content and water absorption capacity at that soaking time. This phenomenon was linked to increase in the rate of extraction of soluble materials of the seeds, due to a higher rate of water uptake, rather than the final amount of water absorbed at a higher temperature^21^.

### Peleg’s rate constant k_1_ and the initial moisture content

From the results of the experiment, it is clear that k_1_ decreased with increase in temperature for each variety (Table 2). This trend is similar to the results of Maskan^24^, Turhan et al.^11^, Solomon^13^, Jideani and Mpotokwana^26^. Therefore, the Peleg rate constant k_1_ is related to the rate of mass transfer^11^ in that its inverse (1/k_1_) is equivalent to the initial hydration rate, as shown by the Peleg’s equation. The sensitivity of k_1_ to temperature is an indication of the positive effect of increasing soaking temperature on the rate and amount of water absorbed^26^, which means there is an increased rate of water absorption at higher temperatures.

### Effects of temperature on Peleg’s rate constant k_1_

The relationship between the reciprocal of k_1_ and temperature was determined using the Arrhenius equation. The result obtained from the linear regression of values of (1/k_1_) and (1/RT) are as stated in the Table 3. From the results, the activation energy (E_a_) values obtained for *M. pruriens*, *M. rajada* and *M. veracruz* are 1631.24, 747.95 and 2743.64 kJ/mol, respectively (Table 3). This suggests that the energy required to trigger water absorption in *M. rajada* is lower, compared to *M. pruriens* and *M. veracruz* and that raising the soaking temperature will have the greatest water absorption improvement effect in *M. veracruz*. This was also the trend in the study of the hydration kinetics of three wheat products of the same variety^22^. Therefore, increasing the soaking temperature for *M. veracruz* can bring about a resultant increase in the rate of water absorption and may tend to reach the equilibrium moisture content at a faster rate.

## Conclusion

Peleg’s equation fitted partly into describing the hydration behaviour of the three varieties of *Mucuna* beans studied (*M. pruriens, M. rajada and M. veracruz*) at 30 °C but the effectiveness of the fit increased with increase in soaking temperature (at 40 °C and 50 °C). Peleg’s rate constant k_1_ decreased with increase in soaking temperature, indicating that water absorption rate increased with increase in temperature. However, Peleg’s capacity constant k_2_ decreased with increase in temperature and as such, the equilibrium moisture content or water absorption capacity decreased with increase in temperature. Arrhenius equation appropriately described the temperature dependence of the water absorption rate and was effective in calculating the activation energies of soaking of each variety of the beans. The study shows that the hydration kinetics of *Mucuna* beans could be predicted and as such, the hydration conditions could be optimized. Practical applications exist for the knowledge of seed physical properties and water uptake kinetics in the design of food processing machines and the strength of materials to be used in their fabrication. The knowledge will also help to design multi-functional processing equipment for seeds that possess closely similar physical and water uptake properties. Future works on the development of regression models that could predict water absorption at lower soaking temperatures are still needed to further understand the kinetics of water uptake under these conditions.
